# A drug eluting poly(trimethylene carbonate)/poly(lactic acid)-reinforced nanocomposite for the functional delivery of osteogenic molecules

**DOI:** 10.2147/IJN.S163219

**Published:** 2018-09-24

**Authors:** Xi Zhang, Mike A Geven, Xinluan Wang, Ling Qin, Dirk W Grijpma, Ton Peijs, David Eglin, Olivier Guillaume, Julien E Gautrot

**Affiliations:** 1 School of Engineering and Materials Science, Queen Mary University of London, Mile End Road, London, UK, j.gautrot@qmul.ac.uk; 2 Institute of Bioengineering, Queen Mary University of London, Mile End Road, London, UK, j.gautrot@qmul.ac.uk; 3 Department of Biomaterials Science and Technology, University of Twente, Enschede, the Netherlands; 4 Translational Medicine R&D Center, Institute of Biomedical and Health Engineering, Shenzhen Institutes of Advanced Technology, Chinese Academy of Sciences, Shenzhen 5018057, China; 5 AO Research Institute Davos, Davos, Switzerland, olivier.guillaume@aofoundation.org

**Keywords:** fiber-reinforced composite, poly(trimethylene carbonate), photo-crosslinking, dexamethasone, osteogenic materials

## Abstract

**Background:**

Poly(trimethylene carbonate) (PTMC) has wide biomedical applications in the field of tissue engineering, due to its biocompatibility and biodegradability features. Its common manufacturing involves photofabrication, such as stereolithography (SLA), which allows the fabrication of complex and controlled structures. Despite the great potential of SLA-fabricated scaffolds, very few examples of PTMC-based drug delivery systems fabricated using photo-fabrication can be found ascribed to light-triggered therapeutics instability, degradation, side reaction, binding to the macromers, etc. These concerns severely restrict the development of SLA-fabricated PTMC structures for drug delivery purposes.

**Methods:**

In this context, we propose here, as a proof of concept, to load a drug model (dexamethasone) into electrospun fibers of poly(lactic acid), and then to integrate these bioactive fibers into the photo-crosslinkable resin of PTMC to produce hybrid films. The hybrid films’ properties and drug release profile were characterized; its biological activity was investigated via bone marrow mesenchymal stem cells culture and differentiation assays.

**Results:**

The polymer/polymer hybrids exhibit improved properties compared with PTMC-only films, in terms of mechanical performance and drug protection from UV denaturation. We further validated that the dexamethasone preserved its biological activity even after photoreaction within the PTMC/poly(lactic acid) hybrid structures by investigating bone marrow mesenchymal stem cells proliferation and osteogenic differentiation.

**Conclusion:**

This study demonstrates the potential of polymer–polymer scaffolds to simultaneously reinforce the mechanical properties of soft matrices and to load sensitive drugs in scaffolds that can be fabricated via additive manufacturing.

## Introduction

Poly(trimethylene carbonate) (PTMC) is a biocompatible and degradable polymeric material that can be synthesized via the ring-opening reaction of 1,3-trimethylene carbonate.^1^ Its degradation, mediated by a surface-erosion mechanism, is characterized by an extremely low level of nonenzymatic hydrolysis and by the release of nonacidic by-products, which make PTMC an attractive material as polyester alternative for medical applications.^2^,^3^ However, PTMC is usually considered to have poor mechanical performance, which restricts its applications, in particular for tissue scaffolding. Several strategies have been developed to improve the mechanical properties of PTMC, by increasing molecular weight,^4^ blending with stiffer polymers or inorganic particles,^5^–^7^ copolymerizing with “hard” polymer blocks,^8^ or crosslinking.^9^ Recently, Schüller-Ravoo et al synthesized three-armed PTMC methacrylate macromers that can be photo-crosslinked to produce flexible and tear-resistant elastomeric materials.^10^ In addition, the ability to photoinitiate crosslinking permits the use of stereolithography (SLA), a common additive manufacturing technique, to build PTMC-based structures with excellent degree of precision in the control of three-dimensional architectures.^11^–^13^

An interesting feature of the slow surface degradation and erosion profile of PTMC-based materials is that they allow good control of the release profile of drugs in the presence of enzymes (such as lipases).^14^–^16^ Despite the potential of SLA-fabricated scaffolds, very few examples of PTMC drug delivery systems fabricated using photofabrication can be found in the literature.^14^ A major inconvenience of SLA, in designing drug-loaded scaffolds, is that UV irradiation and radical generation can result in the degradation or cross-reactivity of the drug being encapsulated, often at relatively low concentrations. Indeed, SLA requires successive layer-by-layer photoreactions of the methacrylate macromers. This intrinsically restricts the potential of SLA-fabricated scaffolds to be used as drug delivery carrier, due to radical-mediated chemical cross-reactions and due to the light sensitivity of the majority of therapeutic compounds. So far, only Vitamin B12 (as model) has been incorporated into PTMC photo-crosslinkable matrix, under the form of nonsoluble microgranules to prevent any degradation.^14^ In order to confer bioactive properties to SLA-fabricated PTMC scaffolds, we recently reported the incorporation of hydroxyapatite (HA) nanoparticles into the PTMC-based photo-crosslinkable resins. The composite PTMC–HA scaffolds successfully stimulated bone formation in a calvarial defect model in rabbit.^11^ Nevertheless, a high loading of HA particles up to 40 weight % was required to elicit beneficial osteogenic effects, which renders the resin highly viscous and difficult to process for SLA-based additive manufacturing. As an alternative, we further developed composite PTMC structures with enhanced mechanical properties, by incorporating electrospun poly(lactic acid) (PLA) fibers in methacrylate-terminated PTMC macromers followed by UV crosslinking.^17^ The improvement in PTMC mechanical performance combined with its potential bioactive and shape memory properties^18^ has brought interest in designing drug delivery systems that can further enhance bioactivity, in particular osteogenicity.

Dexamethasone (Dexa) is an ideal drug candidate for such applications as it is widely used in vitro and in vivo to regulate osteodifferentiation.^19^,^20^ Dexa is a synthetic glucocorticoid with several therapeutic applications, such as antiinflammatory, immunosuppressant, and decongestant.^21^ It also displays potent effects on the proliferation and osteogenic differentiation of mesenchymal stem cells (MSCs) in the presence of β-glycerol phosphate and ascorbic acid/ascorbate,^22^,^23^ with optimal concentrations ranging from 10 to 100 nM.^24^ Similarly, icaritin is a metabolite of the flavonoid glycoside extracted from Herba Epimedii, which was reported to enhance the differentiation and proliferation of osteoblasts.^25^

Various systems have been developed as Dexa carriers, allowing a sustained release, such as implant dispensers,^26^ nanoparticles/hydrogel complexes,^27^ self-assembled nanofibrous gels,^28^ electrospun fibers,^29^ macroporous scaffolds,^30^ and extrusion-based structures.^24^ Among these, Dexa-charged nanoparticles can easily be administered but have a low drug-loading efficiency. It is also difficult to prevent their dispersion in the body following their administration for local treatment. Hydrogel/fiber composites are more suitable for local delivery and showed controlled release profiles, but their poor mechanical performance restricts their potential applications. Electro-spun polymer nanofibers have gained significant interest for drug encapsulation, due to their ease of fabrication, high drug incorporation efficiency, large surface area, and the inherent porosity of the scaffolds they form.^31^ However, a burst release usually occurs as a result of drug accumulation on the fiber surface and the high surface area of these materials.^32^,^33^ The initial burst release can be alleviated by improving drug–polymer compatibility and drug solubility in polymer solution during electrospinning (ie, employing surfactants, although often by compromising cytotoxicity). A sustained release can also be achieved by fabricating complex materials that encapsulate drugs in a core-shell structure, modifying fiber’s chemical properties or using drug-binding agents.^34^–^38^ However, these approaches considerably increase the complexity of the processing methodologies and require novel chemical functionalization that may prevent regulatory approval and clinical translation.

The objective of our work was to fabricate PTMC/PLA nanofiber composite systems (based on fibers with diameters in the micron range and below), allowing the control of the release of model drugs, Dexa and icaritin, promoting osteogenic differentiation in vitro. PLA was selected for the fabrication of electrospun fibers since it is commercially available, biocompatible, degradable, US Food and Drug Administration approved, and significantly stiffer than PTMC (to achieve reinforcement). In addition to composite formulation, PLA has also been routinely used to modify the physical properties of degradable polymers, by copolymerizing with other monomers.^39^ Combination with the PTMC matrix also improves the ductility of brittle PLA fiber materials^17^ and confers shape memory properties, as previously reported by our group.^18^ PLA fibers loaded with Dexa were electrospun and incorporated into a photocured PTMC matrix. In addition to mechanical reinforcement, our results demonstrate the preservation of the drug activity and the control of its diffusion, compared to a direct drug-loaded PTMC strategy. In order to validate this approach, the biological activity of Dexa-loaded PTMC–PLA films was assessed by investigating their osteogenic properties on human MSCs.

The ability to generate mechanically enhanced photocured PTMC composites able to release active therapeutics constitutes an important progress in the use of these materials for additive manufacturing and tissue engineering applications.

## Materials and methods

### Materials

PLA (2002D, 2,000,000 g/mol, density 1.24 g/cm^3^) was obtained from Natureworks. PTMC (three-armed methacrylate-ended, M_n_ 10,000 g/mol) macromer was synthesized as previously reported.^40^ Chloroform, dimethylformamide (DMF), methanol, dichloromethane, ethyl acetate, acidic acid, tetrahydrofuran (THF), and acetonitrile (high-performance liquid chromatography [HPLC] grade) were obtained from Fisher Scientific. Poly(ethylene glycol) methacrylate (average M_n_360 g/mol), dexamethasone (Dexa), 2-Hydroxy-4′-(2-hydroxyethoxy)–2-methylpropiophenone (Irgacure 2959, I2959), and triethylamine were purchased from Sigma-Aldrich. Icaritin was obtained from Shenzhen Institutes of Advanced Technology, Chinese Academy of Sciences. PBS was prepared by dissolving one tablet (Sigma-Aldrich) in 200 mL deionized (DI) water. All materials and reagents were used as received.

### Electrospinning and composites preparation

The electrospinning was performed using an in-house-built electrospinning system. To spin PLA and icaritin/Dexa-loaded PLA fibers, PLA solution at a concentration of 9 wt% in chloroform/DMF (chloroform/DMF =3/1) was first prepared. For icaritin-loaded/low Dexa-loading fibers, 0.50 wt% of icaritin/0.68 wt% of Dexa (with respect to PLA) was added to the PLA solution, respectively. They were stirred until fully dissolved. For higher Dexa-loaded fibers (2.42 wt% with respect to PLA), methanol was used to replace DMF while preparing PLA solution in order to increase the drug solubility. The ratio of chloroform/methanol was set at 3/1. The PLA or PLA-Dexa solution was supplied through a PTFE tube at 1.0 mL/hour to the electrospinning spinneret. The spinning was carried out at a voltage of 18–20 kV and a distance of 15 cm. Random fiber mats were collected on a grounded aluminum foil sheet. Finally, four different fiber mats (neat PLA fiber mats [PLA 0], PLA-icaritin fiber mats, PLA-low Dexa fiber mats [PLA 1], and PLA-high Dexa fiber mats [PLA 2]) were obtained and they were evaporated in vacuum desiccator for 48 hours to remove residual solvent.

A hot press was used to incorporate electrospun fibers into the PTMC matrix. To perform hot pressing, PTMC macromer was first dissolved in dichloromethane at 50 wt% concentration together with 0.67 wt% of Irgacure 2959 (I2959, low cytotoxicity photoinitiator,^41^ with respect to PTMC). After dissolution, the PTMC/dichloromethane solution was transferred into a vacuum desiccator for 48 hours to remove the dichloromethane. The dried PTMC/I2959 mixture was then ready to use for hot pressing (Collin P300E). Forty milligrams of PLA fiber mats with a size of 50×50×0.08 mm, called PLA 0, PLA 1, and PLA 2, for PLA loaded with 0, 0.68, and 2.42 wt% of Dexa respectively, were placed in a 60×60×0.15 mm mold, which was then transferred into the hot press. One hundred sixty milligrams of PTMC macromer (containing I2959) was placed on top of the fiber mat. Fibers and PTMC were prewarmed at 60°C for 5 minutes and then pressed under 25 bar pressure. A compressed film of 0.06 mm thickness was removed from the mold after cooling to room temperature. This was followed by exposing the resulting composite films under UV irradiation (Omnicure 1500) for 100 seconds at 15 mW/cm^2^ to cure the PTMC macromer. The composite films were then stored in a nitrogen box prior to further characterization and experiments. For comparison, PTMC samples without fibers were prepared by casting PTMC/I2959 dichloromethane solution in 50×5×1 mm mold followed by evaporating solvent. PTMC was UV-cured using the same parameters as described above. Four different groups of samples were prepared, namely PTMC (for PTMC without any integrated PLA fiber), PTMC/PLA 0, PTMC/PLA 1, and PTMC/PLA 2 for composite PTMC matrix integrating PLA fibers loaded with 0, 0.68, and 2.42 wt% of Dexa, respectively.

### Electrospun fiber and PTMC/fiber composites characterization

The morphology of electrospun fibers and PTMC/PLA fiber composites was characterized using scanning electron microscopy (SEM, FEI Inspect F). All samples were mounted onto SEM specimen stubs and sputter coated with a thin gold layer for contrast. To investigate PTMC infiltration and PTMC–fiber interaction, composites samples were cold-fractured in liquid nitrogen, and the fracture surface was characterized using SEM. Thermal properties of electrospun fibers were measured using differential scanning calorimetry (DSC, PerkinElmer DSC 4000). Samples were first equili-brated at 25°C and then ramped to 180°C at 10°C/min. Glass transition temperature (*T*_g_) was determined by the midpoint of glass transition. The crystallinity of PLA was calculated by $X_{c}=\frac{H_{m}}{H_{\text{ref}}}\times 100\%$, where *X*_c_ is the crystallinity, *ΔH*_m_ is the experimental heat of fusion at melting point determined by DSC, *ΔH*_ref_ is the theoretical heat of fusion of fully crystalline PLA (93 J/g).^42^ Tensile tests of electrospun fiber mat and PTMC/PLA fiber composites were performed using dynamic mechanical test (TA Q800) in a controlled force mode. Samples were cut into 20×5×0.06 mm rectangular strips before being mounted onto the clamps. A preload force of 0.05 N was applied, and specimens were stretched at 0.1 N/min rate until failure at room temperature. Young’s modulus was derived from the slope of stress–strain curve at low strain (2%), and three tests were performed on each sample.

### Gel permeation chromatography analyses

Gel permeation chromatography (GPC) was employed to investigate the possible coupling between Dexa and the methacrylate-ended PTMC macromers, in the presence of photoinitiator, during UV curing. We examined the refractive index (RI) signal of methacrylate and Dexa before and after UV curing. Instead of PTMC-methacrylate, a model of macromer PEG-methacrylate was used and dissolved in THF at a concentration of 2.0 mg/mL. Dexa and I2959 were added at 0.20 and 0.17 mg/mL, respectively. The solution was degassed with nitrogen for 30 minutes and divided into two portions, one of which was UV treated at 15 mW/cm^2^ for 100 seconds and the other not. For reference, THF solutions of Dexa and I2959 were also tested separately to identify their signals. The GPC tests were performed using Agilent Technologies 1260 Infinity equipped with a UV detector at 308 nm wavelength. THF (with 2.0 vol% triethylamine) was used as the eluent at a flow rate of 1.0 mL/min.

### In vitro measurement of icaritin and dexamethasone release

The amount of icaritin released in PBS was calculated by measuring the remaining icaritin in samples using HPLC (Waters e2695) equipped with the UV detector and a Kinetex column (Phenomenex, C18, 100 Å, 5 µm, 150×4.6 mm). The mobile phase was 25/75 (v/v) water/acetonitrile, eluted at 1.0 mL/min, with water phase adjusted to pH 4 by adding 0.5 vol% of acetic acid and 0.3 vol% triethylamine. The test was performed at 360 nm UV adsorption wavelength with a 20 µL injection. Electrospun PLA fiber mats were cut into 10×10 mm specimens and immersed in 10 mL PBS. At different time intervals, specimens were removed from PBS, rinsed with DI water, and extracted using ethyl acetate. The extractant (ethyl acetate) was evaporated and redissolved in methanol and analyzed using HPLC (n=3 per group). Dexa concentration in PBS was analyzed using the same HPLC equipment described above. The mobile phase was 50/50 (v/v) acetonitrile/PBS, eluted at 1.0 mL/min. The test was performed at 256 nm UV adsorption wavelength with a 50 µL injection for each solution. To evaluate the Dexa elution from electrospun PLA fibers and PTMC/PLA fiber composites, 15×15 mm specimens were cut out from each sample and immersed in 3 mL PBS into an incubator at 37°C for a period of 5 weeks. At different time points, each specimen was removed from the media and transferred into 3 mL fresh PBS. The recovered media was then analyzed using HPLC (n=3 per group).

### Cell culture and differentiation assays

PTMC/PLA fiber composite films (PTMC/PLA 0, 1, and 2) were punched into discs with a diameter of around 6.0 mm (each disk weighs around 1.5 mg). The disks were then placed in 96-well plate and sterilized in ethanol 70% for 10 minutes.

Human bone marrow mesenchymal stem cells (hBMSCs) were isolated from vertebral body bone marrow aspirates and obtained from donors undergoing spinal fusion with informed written consent and full ethical approval (from Kantonale Ethikkommission Bern 126/03). hBMSCs of two donors were expanded individually and seeded separately, at passage 3, onto the films at a density of 20,000 cells/cm^2^. In order to investigate the biological activity of Dexa released from the composite structures (PTMC/PLA 0, 1, and 2), the cells were cultivated in osteogenic media depleted of any Dexa (called “OM−”) based on basic low glucose DMEM (Gibco) supplemented with 10% serum (SeraPlus), 1% penicillin/streptomycin (Gibco), 50 µg/mL ascorbic acid, and 5 mM glycerol-2-phosphate (all from Sigma-Aldrich). The osteogenic differentiation of hBMSCs in the described groups was compared with cells seeded on PTMC/PLA 0, cultivated under nonosteogenic condition (negative control in basal medium, called “BM”, based on basic low glucose DMEM supplemented with 10% serum and 1% penicillin/streptomycin) and complete osteogenic medium (positive control, called “OM+” similar to “OM−” composition but supplemented with 10 nM Dexa, from Sigma-Aldrich). After seeding, the 96-well plates were filled with 200 µL of the different medium and were changed three times a week for the 28 days of the osteogenic experiment. For all the in vitro investigations, control surfaces based on tissue culture polystyrene (TCPS) were used (96-well plate TPP, Trasadingen, Switzerland).

The cytocompatibility of the different composite films and the cell proliferation kinetic was evaluated using Cell-Titer Blue assay (Promega, Dübendorf, Switzerland) at 2, 6, 14, 21, and 28 days post seeding (n=5 per group), following the supplier’s recommendation. The resulting fluorescence intensity was read with a multiplate reader (Viktor,^3^ 1,420 Multilabel Count, Perkin-Elmer) and values were corrected using cell-free condition.

For DNA quantification, samples were first incubated in lysis buffer made of Triton X-100 at 0.1% in 10 mM of Tris-HCl, pH =7.4 (all from Sigma-Aldrich) and followed by one freezing–thawing cycle. Then, DNA amount was estimated using fluorescent CyQuant^®^ GR Dye assay, according to the supplier’s recommendation (Invitrogen, n=3 per group). Alkaline phosphatase (ALP) activity from the cell-lysis solution was determined using colorimetric quantification. Briefly, samples along with a set of standard solutions (*p*-nitrophenol of concentrations from 0 to 1,000 µM) were incubated with alkaline buffer solution (2-amino-2-methylpropanol 1.5 M pH =10.3, from Sigma-Aldrich) and then ALP substrate buffer was added (phosphatase substrate dissolved in diethanolamine buffer at 1 M in 0.5 mM MgCl_2_ adjusted pH =9.8). After mixing and heating (at 37°C for exactly 15 minutes), a solution of NaOH at 0.1 M was added to each tube in order to stop the reaction. Then, the intensity of *p*-nitrophenol formation was monitored at 405 nm. The total ALP contents were expressed as enzyme activity units in nmol/min (n=3 per group), as a function of total DNA (ng) per well measured using CyQuant assay. ALP staining was performed after washing the cell monolayers with PBS (three times), fixation (with ice cold ethanol 90% for 4 minutes), washing with DI water and lately, staining with Fast Blue dye solution for 1 hour wrapped in tin foil (Naphthol AS-MX, according to Sigma’s recommendation). After incubation, samples were washed three times with DI water and imaged by light microscopy (Macrofluo™ from Leica).

The occurrence of mineralization was detected using Alizarin Red Staining (ARS, Sigma-Aldrich), with TCPS used as control films. The cell monolayer was washed with PBS, fixed with formaldehyde 4%, and further washed with DI water. Then, 40 mM ARS solution at pH =4.2 was added to each well for 1 hour and thoroughly washed with DI water for 5 days. Finally, samples were imaged by light microscopy (Macrofluo) and a quantification of the ARS was performed by acid extraction thereafter. Briefly, acetic acid (at 10%) was added to each well for 30 minutes, and the loosely attached monolayer of cells was transferred to Eppendorf tubes and heated up to 85°C for 10 minutes, then placed on ice for 5 minutes. After centrifugation at 20,000 g for 15 minutes, ammonium hydroxide was added to the supernatant (final pH of 4.1–4.5) and absorbance was recorded at 405 nm and compared to ARS standard solutions ranged from 0 up to 2,000 µM (n=3 per group; for all the mentioned assays, background values obtained from cell-free condition are subtracted from the final values).

SEM analyses required the fixation of the samples overnight in buffered paraformaldehyde at 4%, the dehydration with gradual concentration of ethanol up to 100% followed by immersion in hexamethyldisilazane (Sigma-Aldrich). After complete drying, the samples were sputter coated with C and investigated using a Hitachi S4700 Field Emission Scanning Electron Microscope (FESEM) instrument. In order to validate the presence of CaP mineralization by hBMSCs triggered by the release of Dexa from PTMC/PLA films, we carried out energy dispersive X-ray analysis (EDX, Oxford Instruments, Abingdon, UK), following C coating.

### Statistical analyses

Statistical analysis of data was performed using Prism software (GraphPad Software, La Jolla, CA, USA). We assumed normal distribution of data. One-way ANOVA with Tukey’s multiple comparison test was applied to detect significant differences between experimental groups (with *P*<0.05). Data presented are means ± standard deviation (SD) unless stated otherwise.

## Results and discussion

### Physical properties of the PTMC/PLA hybrid structures

**Figure 1:**
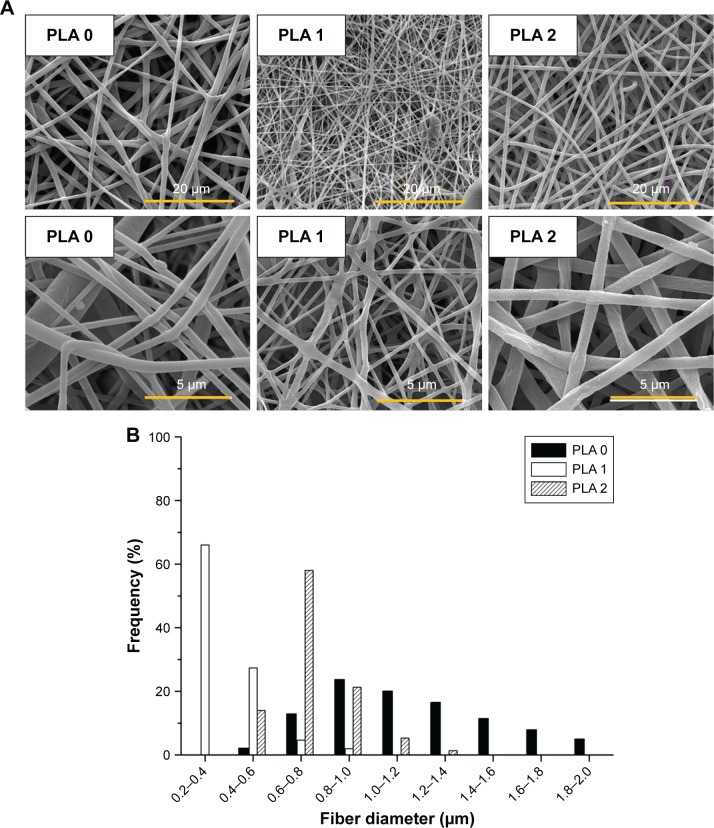
Loading Dexa in PLA results in homogenous and smooth PLA electrospun fibers. **Notes:** (**A**) SEM images of electrospun fibers; (**B**) fiber diameter distribution of PLA 0, PLA 1, and PLA 2. **Abbreviations:** PLA, poly(lactic acid); SEM, scanning electron microscopy.

The morphologies of electrospun fibers (with and without Dexa) were characterized using SEM (Figure 1A). The incorporation of Dexa into PLA produced more uniform and smaller fiber dimensions, with an average diameter for PLA fibers decreasing from 1.23±0.47 µm (PLA 0) to 0.39±0.14 and 0.74±0.15 µm for PLA 1 and PLA 2, respectively (Figure 1B). Some heterogeneity (eg, beads) was observed on fibers with 0.68 wt% Dexa, which is ascribed to the decreased viscosity of the spinning solution. However, no beads were observed on fibers with 2.42 wt% Dexa, which displayed smooth surfaces. The difference was attributed to the increased solubility of Dexa in methanol (used at higher Dexa concentrations) compared with DMF. Moreover, the formation of larger fibers using methanol (fibers with high drug loading) compared with those formed in DMF (fibers with low drug loading) is explained by the faster evaporation rate of methanol compared with DMF, which resulted in quicker solidification of the fluid jet and reduced fiber stretching.

**Figure 2:**
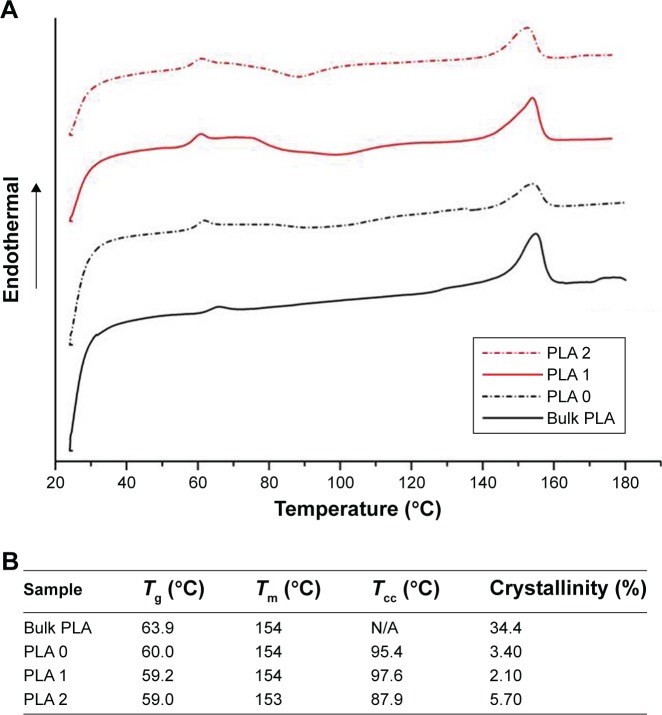
PLA processing and drug loading influence the crystallinity of the electrospun nanofibers. **Notes:** DSC thermograms of the different PLA materials without Dexa (bulk PLA and PLA 0) and with Dexa loading (PLA 1 and 2) (**A**). Quantification of the PLA fibers’ thermal properties, depending on the processing method and the presence of Dexa (**B**). **Abbreviations:** DSC, differential scanning calorimetry; PLA, poly(lactic acid) ; T_g_, glass transition temperature; T_m_, melting temperature; T_cc_, cold-crystallization temperature.

The thermal properties of electrospun fibers were characterized next, using DSC (Figure 2A and B). A melting point (*T*_m_) at 154°C is measured for all samples except for PLA 2 (153°C). The glass transition temperature (*T*_g_) of bulk PLA (63.9°C) was decreased after electrospinning (PLA 0, 60.0°C) and further decreased to 59.2°C (PLA 1) and 59.0°C (PLA 2), respectively, upon incorporation of Dexa. The decrease in *T*_g_ after electrospinning is caused by the inner stress retained within fibers as a result of jet stretching, which makes molecules become mobile at lower temperatures.^32^ Small molecules, such as Dexa, are considered to act as plasticizer and further decreased the *T*_g_ of PLA, although this transition remained significantly higher than body temperature. Cold crystallization is observed on all electrospun PLA fibers’ thermograms. The cold crystallization peak becomes sharper, and cold crystallization temperature (*T*_cc_) is shifted to lower temperatures (from 95.4°C to 87.9°C) after incorporating 2.42 wt% Dexa. The crystallinity of bulk PLA is decreased from 34.4% to 3.40% after electrospinning and further decreased to 2.10% after adding 0.68 wt% Dexa. However, the crystallinity is increased slightly to 5.70% when using methanol instead of DMF. The changes in PLA crystallinity are considered to affect the mechanical properties of fibers which were further studied via tensile tests.

**Figure 3:**
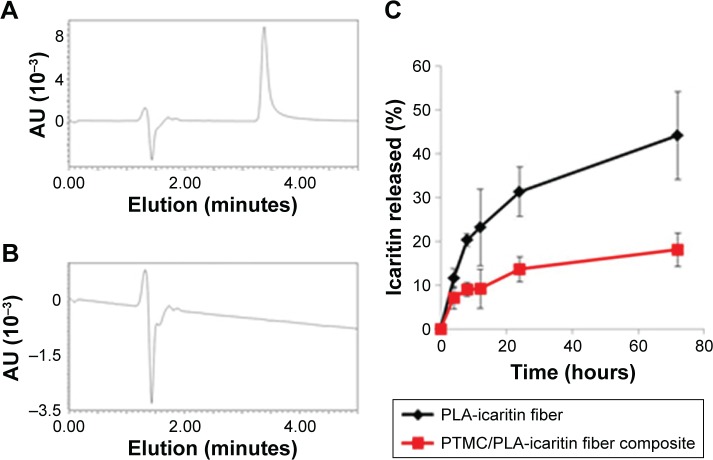
Representative chromatogram of (**A**) direct icaritin injection and (**B**) THF extractant of PTMC/icaritin-loaded fiber composite; (**C**) release profile of icaritin from electrospun fiber (black) and hot-pressed PTMC/PLA fiber composite (red). **Abbreviations:** PLA, poly(lactic acid); PTMC, poly(trimethylene carbonate); THF, tetrahydrofuran.

We next investigated the formation of PTMC/PLA fiber composites. In our previous report,^17^,^18^ PTMC/PLA fiber composites were prepared by impregnating PTMC/propylene carbonate solution into electrospun PLA fiber mat, followed by UV crosslink and solvent extraction (for removing propylene carbonate). Based on the established method, we first integrated icaritin-loaded PLA fibers into PTMC and monitored its in vitro release, via HPLC. A representative chromatogram of direct icaritin injection is shown in Figure 3A; an icaritin peak at 3.5 minutes elution time was observed. However, in the following in vitro release assays, no icaritin release was observed from PTMC/PLA fiber composites in contrast to the slow release of icaritin observed from PLA fibers alone. To verify whether icaritin was physically trapped in PTMC or lost during composite preparation procedure, a series of tests were performed. Firstly, the PTMC/icaritin-loaded fiber composites were incubated in a good solvent, THF, for 24 hours. The supernatant was retrieved and analyzed using HPLC; no icaritin was detected (a representative chromatogram of supernatant is shown in Figure 3B, in which the icaritin peak disappeared). Secondly, PTMC/PLA fiber composites were incubated in icaritin/THF solution at known concentration for 24 hours. No decrease in icaritin concentration was observed, implying that icaritin does not simply remain trapped in these scaffolds. Thirdly, icaritin was incorporated into PTMC by directly dissolving in PTMC/propylene carbonate solutions. The icaritin-loaded PTMC was extracted by THF, but still no icaritin was observed by HPLC. Hence, our data suggested that icaritin was not physically trapped in the composites but was somehow degraded or trapped during the photopolymerization process. We further analyzed the extracted fractions that were used for the removal of propylene carbonate after polymerization. No icaritin was detected in these solutions either, indicating that no icaritin was lost during the composite preparation procedure. We therefore proposed that icaritin was extracted from electrospun fibers into the PTMC/propylene carbonate phase and reacted with methacrylate-ended PTMC macromers during the UV crosslinking of the matrix. Hot pressing, a solvent-free composite preparation method, was therefore used for the rest of our work. Indeed, after icaritin-loaded electrospun PLA fibers were incorporated into PTMC, icaritin release was observed and monitored by HPLC (Figure 3C). Therefore, in these conditions, PLA fibers act as a protecting phase for icaritin loading.

**Figure 4:**
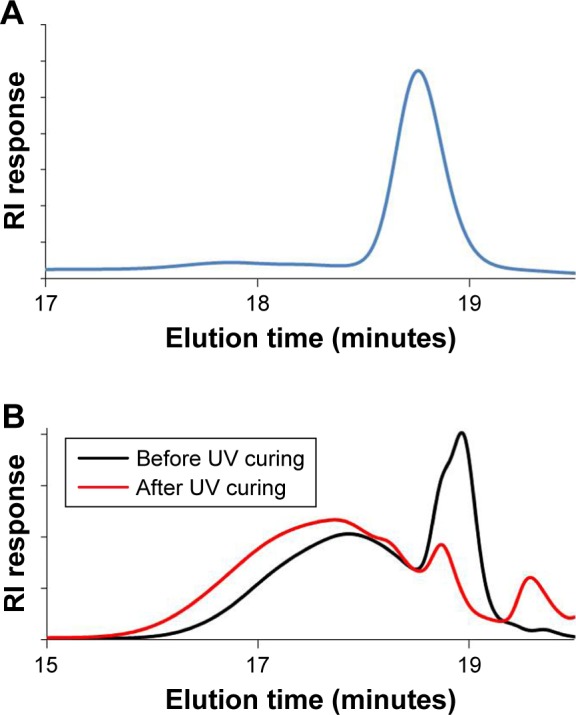
If unprotected, Dexa reacts with methacrylated macromeres during UV reaction. **Notes:** RI signal against elution time of dexamethasone (**A**); RI signal against elution time of Dexa and PEG-methacrylate before (black) and after (red) UV curing (**B**). **Abbreviation:** RI, refractive index.

The versatility of this approach for the encapsulation of various drugs into UV-crosslinked PTMC/PLA fiber composites was further demonstrated by replacing icaritin with Dexa. To investigate the occurrence of cross-reactions between methacrylate end groups and Dexa, a PEG methacrylate was photocured in the presence of Dexa. The molar ratio of methacrylate: Dexa: PI was set at 10:1:1, comparing to that in PTMC composite where methacrylate: Dexa: PI was 10:0.15:1 (for PTMC/PLA 1) and 10:0.5:1 (for PTMC/PLA 2), respectively. Higher Dexa concentration was used to increase signal intensity. The starting materials and resulting products were characterized via GPC. Dexa was identified by its RI signal, at 18.9 minutes (Figure 4A). The RI signal against elution time of Dexa and PEG-methacrylate before and after UV curing is presented in Figure 4B. After UV curing, the intensity of Dexa peak (eluted at 18.9 minutes) is significantly reduced. In the meantime, the peak corresponding to PEG-methacrylate (17.9 minutes) is shifted to a lower elution time (17.7 minutes) with increased signal intensity. The shift of PEG-methacrylate signal peak, the decreased signal intensity of Dexa, and the increased signal intensity of PEG-methacrylate constitute further evidence for the coupling between Dexa and PEG-methacrylate. The reaction between Dexa and methacrylate-ended macromers shows the instability of Dexa when exposed to UV-curing processes and its potential deactivation when directly integrated into methacrylate-based matrices. It is therefore crucial to protect such drugs from exposure of radicals involved in the curing reaction.

**Figure 5:**
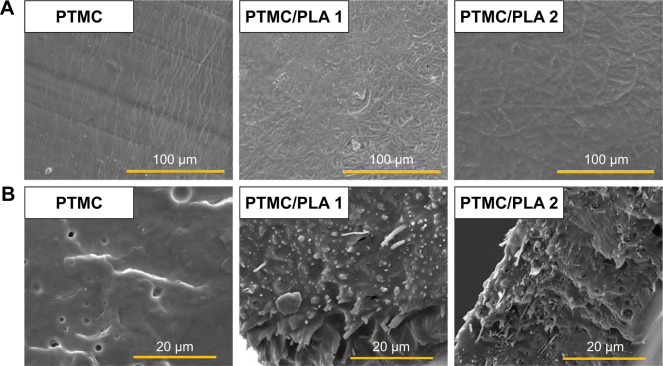
PLA nanofibers exhibit a good physical interaction in hybrid PTMC/PLA structures. **Note:** SEM images of (**A**) sample’s surface and (**B**) sample’s cross-section. **Abbreviations:** PLA, poly(lactic acid); PTMC, poly(trimethylene carbonate); SEM, scanning electron microscopy.

Therefore, we proposed to use PLA electrospun fibers hot pressed into a PTMC matrix to protect Dexa loaded during photo-crosslinking, to ensure drug’s retention and release from the resulting scaffolds under its active form. SEM images of PTMC/PLA fiber composites are presented in Figure 5, where both sample surface and cross-sections are presented. The composite surfaces are covered by PTMC, with some PLA fibers exposed. We observed a good compatibility of the composite structures, as PLA fibers are well wetted by the PTMC matrix, and interspaces between fibers are filled by the matrix resulting in nearly void-free composites (Figure 5). Strong interfacial bonding of PLA fibers to PTMC is evidenced by SEM as most fibers remain well embedded within the matrix upon fracture of the corresponding samples, indicating good levels of interactions between fibers and the surrounding PTMC matrix.

**Figure 6:**
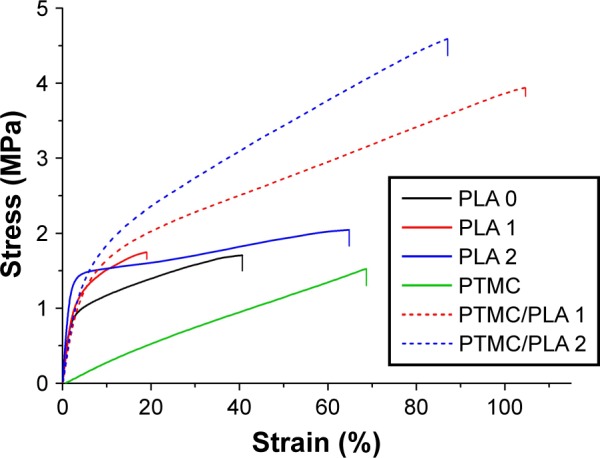
Incorporation of PLA nanofibers into PTMC films dramatically improves the mechanical resistance of materials. **Note:** Representative stress–strain curve of electrospun fiber mat and PTMC/PLA fiber composites. **Abbreviations:** PLA, poly(lactic acid); PTMC, poly(trimethylene carbonate).

The mechanical properties of both electrospun fibers and PTMC/PLA fiber composites are quantified, and the results are presented in Figure 6 and Table 1. It is found that Young’s modulus and strength of electrospun fiber mats are much lower compared with bulk PLA (3.5 GPa, provided by supplier), although electrospun nanofibers (diameter 200–300 nm) were reported to exhibit Young’s moduli up to three times that of bulk PLA.^43^ The reason for this decrease is the combined effect of the porosity of the mats and the lack of orientation of the fibers, allowing fiber–fiber sliding and reorientation during stretching of the mats. PLA 2 displays a higher Young’s modulus than PLA 0, presumably due to its higher crystallinity (5.7% compared with 3.4%). Meanwhile, PLA 1 exhibits lower failure strain, which may be explained by its more heterogeneous structure, with the presence of beads in the nanofibers (see Figure 1A, sample PLA 1), which can act as potential defects, resulting in lower failure strains. However, the mechanical properties of PTMC were significantly improved by the addition of electrospun PLA fibers. Young’s moduli of PTMC composites increased by more than one order of magnitude, compared with the simple PTMC matrix, and their tensile strength increased by three- to fourfolds. This is an indication of the high reinforcing efficiency of the PLA fibers, as a result of the good integration of the electrospun fibers in the PTMC matrix.

### In vitro release of dexa from electrospun fiber and PTMC/fiber composites

**Figure 7:**
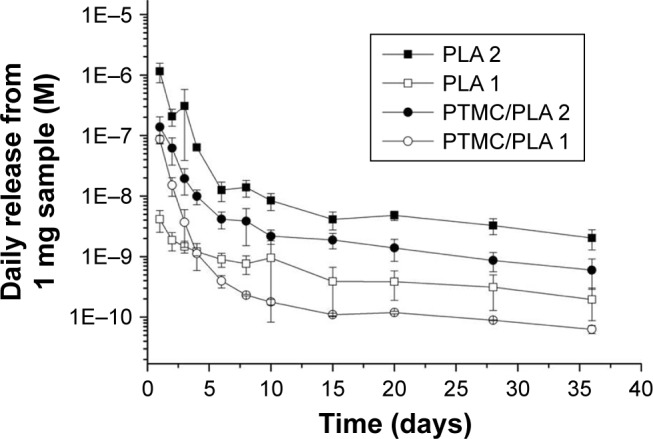
Hybrid films are characterized by a sustained and prolonged release of Dexa. **Notes:** Dexamethasone concentrations released daily from electrospun fibers and PTMC/PLA fiber composites (per 1.0 mg sample in 1.0 mL PBS at 37°C, values presented are noncumulative). **Abbreviations:** PLA, poly(lactic acid); PTMC, poly(trimethylene carbonate).

The in vitro release profile of Dexa from electrospun fibers and PTMC/PLA fiber composites was examined next, over a period of 5 weeks, via HPLC analysis of the supernatant (Figure 7). All samples showed a quick decrease in their release rate in the first 8 days followed by a stable and sustained release profile. PLA 2 exhibited the fastest release rate over the whole test period compared with other samples (initially 1.1×10^−6^ M/day then decreased to 2.0×10^−9^ M/day after 5 weeks). By incorporating Dexa into PTMC composites, the elution kinetic is effectively reduced by 6–10 folds in the first 4 days. In comparison, a more stable Dexa release rate is achieved by incorporating PLA 2 into PTMC, for which release concentrations ranged from 1.4×10^−7^ M/day to 6.0×10^−10^ M/day. PLA 1 fibers displayed the slowest initial release but a stable release profile, ranging from 4.1×10^−9^ M/day to 2.0×10^−10^ M/day. After integrating them within PTMC, the composites exhibited a faster initial release rate than fibers alone, in the first 3 days, but a stable release was maintained after 10 days. The rapid initial Dexa release from composites is ascribed to the incomplete coverage of PTMC on the fiber surface (see Figure 3).

For in vitro cell assays (4 weeks period), PTMC/PLA fiber composites were used (both low and high Dexa loading [PTMC/PLA 1 and PTMC/PLA 2], compared to drug-free PTMC/PLA 0). According to the release kinetic results obtained (Figure 7), we can extrapolate that the concentrations of Dexa released in the culture media (using composite discs of 1.5 mg incubated in 200 µL cell culture medium) will range between 9.4×10^−7^ M and 6.5×10^−9^ M for PTMC/PLA 2 and 6.4×10^−7^ M and 6.7×10^−10^ M for PTMC/PLA 1, which are in the bioactive concentration windows as previously mentioned.^24^

In addition, after 5 weeks of incubation in PBS, the PTMC/PLA fiber composites were characterized using SEM (see Supplementary material Figure S1). These images clearly indicate that the composite structures were well preserved, with similar features to those initially observed on pristine composites (Figure 5), for both surface and cross-section analyses. We observed that the PLA fibers were still fully embedded within the PTMC matrix, indicating that the hybrid PTMC/PLA fiber structures are morphologically stable during the 5 weeks of experiment.

### In vitro differentiation of MSCs triggered by Dexa release from composites

Having confirmed the ability to release Dexa from PTMC/PLA composites, we next examined their potential to be used as a carrier of Dexa and to maintain its bioactivity to trigger osteogenic differentiation of MSCs. To this aim, Dexa was selected as drug model has it exhibits a strong concentration-dependent biological activity on stem cells. For instance, depending on the charge of Dexa in medium, it can favor in vitro hBMSCs proliferation and/or osteogenic differentiation. Both et al showed that cell culture medium supplemented with 10^−8^ M of Dexa promoted both the proliferation and the differentiation of hBMSCs.^44^ However, a reverse effect on hBMSCs has been reported using higher Dexa dosages (ie, 10^−7^ M), with a shift toward adipogenic differentiation associated with a decrease in cell proliferation rate.^44^–^46^ Such a biological activity makes Dexa an excellent candidate to validate the control of the release of Dexa enabled by PTMC/PLA hybrid systems.

**Figure 8:**
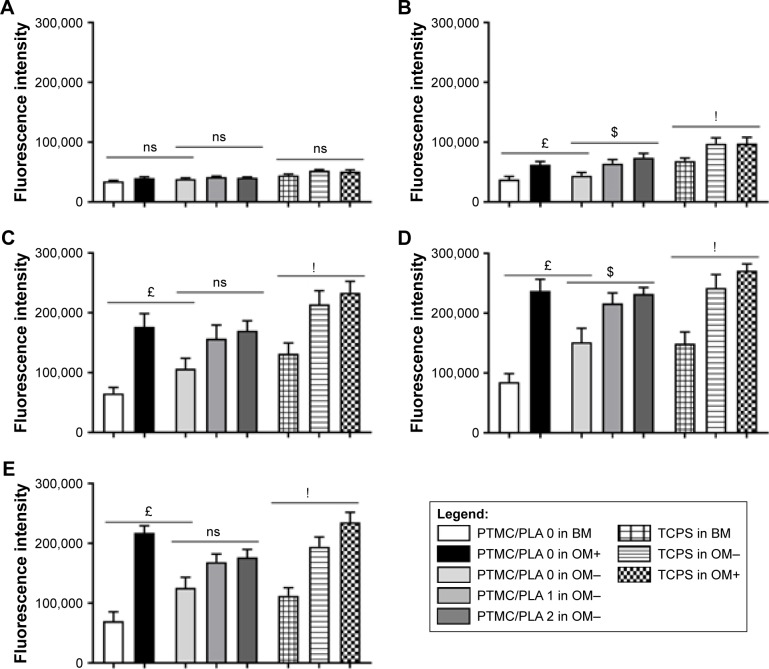
Dexa released from PTMC/PLA composite films impacts on hBMSCs proliferation. **Notes:** CellTiter Blue quantification of hBMSCs proliferating on the different substrates in various media (BM, OM−, and OM+) on Day 2 (**A**), Day 6 (**B**), Day 14 (**C**), Day 21 (**D**), and Day 28 (**E**). £ reports significance for drug-free PTMC/PLA 0 regarding the nature of the medium, $ reports significance for drug-loaded PTMC/PLA on OM− medium, and ! reports significance for TCPS regarding the nature of the medium. ns reports nonsignificance. **Abbreviations:** BM, basal medium; hBMSCs, human bone marrow mesenchymal stem cells; OM, osteogenic media; PLA, poly(lactic acid); PTMC, poly(trimethylene carbonate); TCPS, tissue culture polystyrene.

Two days post seeding (Figure 8A), no difference in hBMSCs density could be detected between the different groups containing Dexa or not, either in the media or loaded in the films. Indeed, when cells were seeded at a low density of 6,000 cells/well, a 3–5 days lag phase was usually observed, before a rapid growth phase is resumed.^44^ This corroborates our results as the effect of Dexa could be first seen at Day 6 (Figure 8B), with a significant increase in cell proliferation for all groups containing Dexa. The effective release of Dexa from PTMC/PLA 1 and 2 therefore correlates with an accelerated cell growth for those two groups in comparison to Dexa-free PTMC/PLA 0, in media depleted of any Dexa (OM−), therefore confirming the retention of the activity of DM upon elution from PTMC/PLA scaffolds. Similar conclusions can be drawn for the later time points (Days 14, 21, and 28, Figure 8C and D, and E respectively), with superior hBMSCs proliferation on OM+ condition, observed on both PTMC/PLA 0 and controls TCPS, and on PTMC/PLA loaded with Dexa (1 and 2) compared with PTMC/PLA 0 even if not systematically significant. At Day 21, fluorescence values for the PTMC/PLA 0 and TCPS groups cultivated in OM+ were similar to the PTMC/PLA 1 and 2, demonstrating the beneficial effect of Dexa released from the composite films as no significance was observed between PTMC/PLA 0 and TCPS in OM+ compared with PTMC/PLA 1 and PTMC/PLA 2. As the cells reached high degrees of confluency following 21 days of cultivation, the fluorescent values measured at Day 28 were not increased compared with previous time points, but results displayed similar trends (Figure 8E). For all time points, cell proliferation was always the lowest in BM conditions, in agreement with previous results, showing that ascorbic acid is an important stimulator of hBMSCs proliferation, in addition to Dexa.^47^

The quantification of DNA conducted Day 21 (Figure S2) reflects the beneficial effect of Dexa released from PTMC/PLA 1 and 2 on hBMSCs proliferation, as previously described.

Overall, our results demonstrated that the sustained release of biologically active Dexa from PTMC/PLA scaffolds stimulates hBMSCs proliferation, but without following a concentration-dependent scenario, as values for PTMC/PLA 1 were similar to PTMC/PLA 2 for all time points. Indeed, as shown by the release kinetic presented in Figure 7, the composite structures with the Dexa charges of 0.68 and 2.42 wt% release the drug at a concentration below the cytotoxic threshold of 10^−7^ M.^45^,^46^

**Figure 9:**
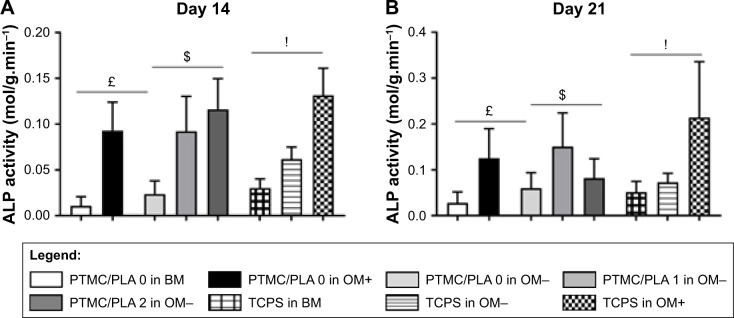

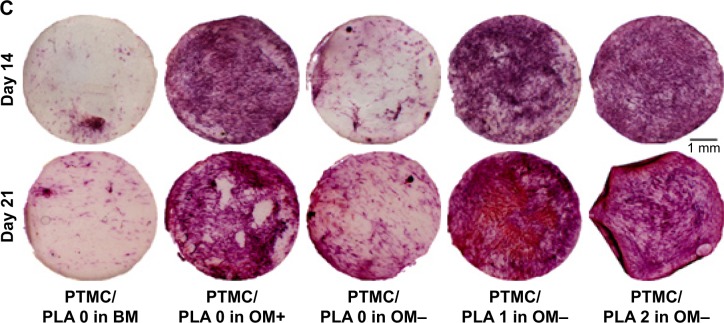
Dexa released from hybrid PTMC/PLA film stimulates ALP activity, early marker of hBMSCs osteogenic differentiation. **Notes:** ALP activity measured on Days 14 and 21 (**A** and **B** respectively, £ reports significance for drug-free PTMC/PLA 0 regarding the nature of the medium, $ reports significance for drug-loaded PTMC/PLA on OM− medium, and ! reports significance for TCPS regarding the nature of the medium). ALP staining on hBMSCs monolayer cultivated on the diverse substrates is shown (for only one donor, but similar staining was obtained for both donors, **C**). **Abbreviations:** ALP, alkaline phosphatase; hBMSCs, human bone marrow mesenchymal stem cells; OM, osteogenic media; PLA, poly(lactic acid); PTMC, poly(trimethylene carbonate); TCPS, tissue culture polystyrene.

It is well known that supplementing media with the synthetic glucocorticoid Dexa at an appropriate dosage induces hBMSCs to differentiate toward osteogenic lineage. One early biochemical marker commonly investigated to validate osteogenic differentiation in vitro is ALP.^48^,^49^ For both time points investigated (Days 14 and 21, Figure 9A and B, respectively), the ALP activity was negligible on BM condition on both PTMC/PLA 0 and TCPS and on OM− without Dexa. Robust ALP stainings were observed in OM+ conditions, but also for cells growing on PTMC/PLA 1 and 2 substrates (Figure 9C, shown for only donor 1). Hence, the continuous release of Dexa from hybrid films triggers stem cells differentiation, to a similar level to that observed for cells cultured in OM+, where media were constantly refreshed with 10 nM of Dexa, as no significant difference was measured between PTMC/PLA and TCPS in OM+ and PTMC/PLA 1 and 2.

As ALP is an early osteogenic marker, it is not surprising to observe a decline of its activity between Day 14 and Day 21 for some groups (eg, PTMC/PLA 2), revealing that the peak of ALP activity has already passed between these two time points. In fact, as PTMC/PLA 2 releases more Dexa than PTMC/PLA 1 (Figure 7), it is reasonable to hypothesize that the ALP peak has occurred earlier in this condition and that, at Day 21, the ALP activity has already decreased again. Such a phenomenon was reported in other studies, as during cell maturation ALP naturally decreases and cells start to deposit minerals (calcium and phosphate), considered as a later marker of osteogenic differentiation.^50^ In vitro mineralization was monitored in our study using ARS and quantification.

**Figure 10:**
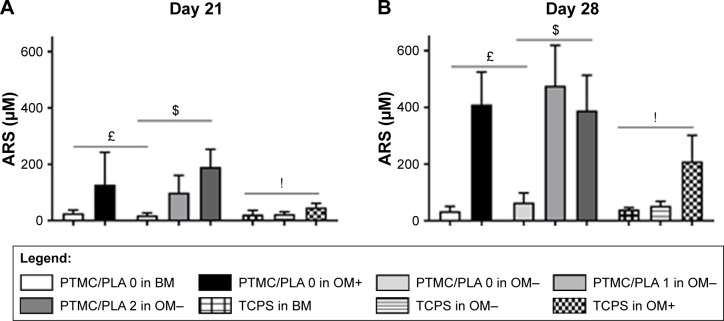

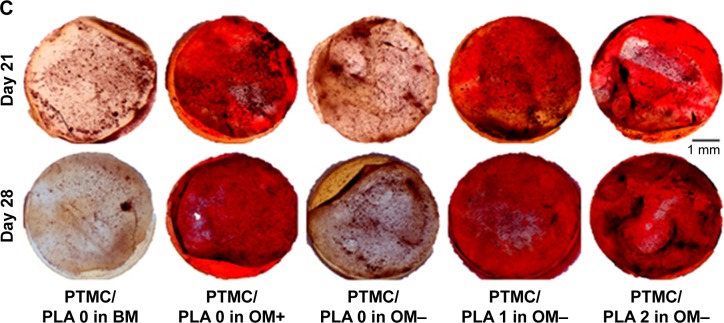
Dexa-loaded PTMC/PLA film successfully triggers hBMSCs differentiation toward mineralizing osteoblast-cell lineage. **Notes:** Calcium deposition from hBMSCs was measured on Days 21 and 28 (**A** and **B** respectively, £ reports significance for drug-free PTMC/PLA 0 regarding the nature of the medium, $ reports significance for drug-loaded PTMC/PLA on OM− medium, and ! reports significance for TCPS regarding the nature of the medium). ARS of Ca^2+^ secreted by hBMSCs cultivated on the diverse substrates is shown (for only one donor, but similar staining was obtained for both donors, **C**). **Abbreviations:** ARS, Alizarin Red Staining; BM, basal medium; hBMSCs, human bone marrow mesenchymal stem cells; OM, osteogenic media; PLA, poly(lactic acid); PTMC, poly(trimethylene carbonate); TCPS, tissue culture polystyrene.

Further indication of osteogenic differentiation of hBMSCs induced by the release of Dexa was evidenced by the staining and quantification of calcium deposition (Figure 10). At both the time points investigated (Days 21 and 28), the Dexa-depleted medium, present in the BM and drug-free Dexa PTMC/PLA 0 in OM− conditions, did not permit cells to mineralize their matrix (Figure 10A and B). In contrast, the presence of Dexa either directly supplemented within the medium (in OM+) or diffusing from PTMC/PLA 1 and 2 scaffolds allowed hBMSCs to fully undergo osteogenic differentiation with robust time-dependent biomineralization (ARS images, Figure 10C). No significance was observed between Ca^2+^ formed in fully supplemented OM media and in the PTMC/PLA 1 and 2 for both time points. In this study, Dexa was selected as a driving source model for osteogenic differentiation. For the positive controls, this factor was directly introduced via the culture medium of hBMSCs (OM+).

This osteogenic study therefore demonstrates that hybrid PLA/PTMC films loaded with Dexa successfully trigger hBMSCs differentiation toward a mature osteoblast lineage, as both early (ALP activity, Figure 9) and late (Ca^2+^ deposition, Figure 10) markers were upregulated to similar levels to those observed for the positive OM+ condition.

**Figure 11:**
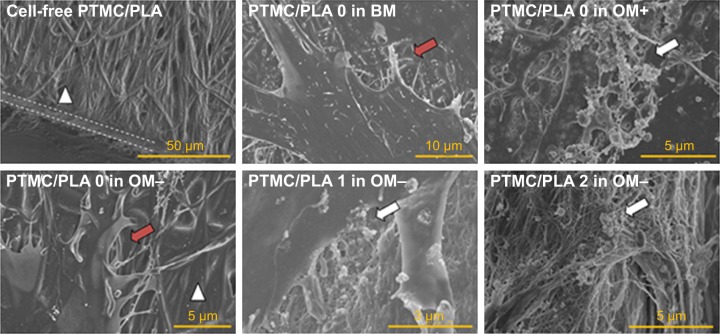
Biomineralization is visible on cell monolayers cultivated on Dexa-loaded films like in OM+ condition. Illustration of PTMC/PLA composite film surface (dashed lines denote the cross-section and white triangle the PLA fibers). **Notes:** The red and white arrows denote cells’ membrane and clusters of minerals, respectively. SEM was realized on Day 28 of the in vitro culture experiment. **Abbreviations:** OM, osteogenic media; PLA, poly(lactic acid); PTMC, poly(trimethylene carbonate); SEM, scanning electron microscopy.

In addition, SEM of samples obtained after 28 days of cell culture (Figure 11) corroborated ARS results. We could not detect any clusters of minerals deposited by the hBMSCs on the control groups (cells cultivated in the absence of Dexa, ie, PTMC/PLA 0 in BM and in OM−), whereas numerous inorganic clusters (supposedly CaP) could be distinguished for the positive control (OM+) and on Dexa-loaded composite films (PTMC/PLA 1 and 2). Further EDX analyses confirmed the presence of Ca and P elements in the pericellular regions of hBMSCs cultivated on Dexa-loaded film (Figure S3). Therefore, SEM images confirm the potential of Dexa-loaded PTMC/PLA composite films to stimulate stem cells differentiation and to promote the deposition of minerals, essential for the application of these matrices in bone tissue engineering.

## Conclusion

In this study, biocompatible and degradable polymeric composites based on electrospun PLA fibers and photo-crosslinked PTMC were successfully fabricated. The fibers were incorporated into PTMC macromer using a hot-pressing method followed by UV curing. The composites exhibited significant improvements in mechanical performance compared with neat PTMC. The incorporation of PLA fibers increases the PTMC’s Young’s modulus by one order of magnitude and its tensile strength by threefolds. The PLA fibers showed strong interfacial bonding with PTMC matrix (no fiber pull-out was observed for cold-fractured composites) and physical stability was observed, even after 5 weeks of in vitro incubation. Dexa was loaded into composites by first co-electrospinning with PLA and then integration into the PTMC matrix. Using this approach, the UV-triggered cross-reaction between Dexa and methacrylate-terminated PTMC macromers was avoided. Thus, the biological activity of Dexa integrated in such a polymer–polymer composite structure was preserved. Moreover, the combination of electrospun fibers with PTMC matrix also achieved a stable and sustained Dexa release profile, which allowed the improvement of hBMSCs proliferation and osteogenic differentiation. Overall, the concept of polymer/polymer hybrid structures offers a high degree of versatility as various therapeutics, especially those known to react with photo-crosslinking reaction, can be loaded in the corresponding scaffolds. This study demonstrates the potential of polymer–polymer scaffolds to simultaneously reinforce the mechanical properties of soft matrices and to load sensitive drugs in scaffolds that can be fabricated via additive manufacturing.

## Supplementary materials

Figure S1 SEM images of PTMC/PLA fiber composites after 35 days in vitro release tests: PTMC/PLA 1 (**A1**) surface, (**A2**) cross-section and PTMC/PLA 2 (**B1**) surface, (**B2**) cross-section (scale bar 20 µm).
**Abbreviations:** PLA, poly(lactic acid); PTMC, poly(trimethylene carbonate); SEM, scanning electron microscopy.

Figure S2 Cell quantification determined by DNA measurement of hBMSCs present on the different substrates in various media (BM, OM− and OM+) at Day 21, for two donors presented separately (in ng/film).
**Abbreviations:** BM, basal medium; hBMSCs, human bone marrow mesenchymal stem cells; OM, osteogenic media; PLA, poly(lactic acid); PTMC, poly(trimethylene carbonate); TCPS, tissue culture polystyrene.

Figure S3 EDX analysis of biomineralization illustrated on sample PTMC/PLA 2 in OM− revealing the presence of Ca and P elements deposited in the pericellular environment (square) and its absence on cell-free area (triangle). This analysis was determined by energy-dispersive X-ray (EDX, Oxford Instruments, Abingdon, UK), following C coating.
**Abbreviations:** EDX, energy dispersive X-ray; OM, osteogenic media; PLA, poly(lactic acid); PTMC, poly(trimethylene carbonate).

## Figures and Tables

**Table 1:** Results of the stress–strain test of electrospun fiber mats and PTMC/PLA fiber composites

Sample	Young’s modulus (MPa)	Strength (MPa)	Failure strain (%)
PLA 0	45.32±5.45	2.31±0.94	50.42±27.23
PLA 1	49.66±5.30	2.14±0.36	20.68±4.61
PLA 2	65.89±21.98	2.18±0.82	72.34±6.85
PTMC	2.73±0.48	1.31±0.43	62.17±11.42
PTMC/PLA 1	30.92±6.80	4.61±1.19	106.85±9.78
PTMC/PLA 2	33.96±19.33	3.86±1.41	81.97±13.17

**Abbreviations:** PLA, poly(lactic acid); PTMC, poly(trimethylene carbonate).
