# Supplementary figures and images for: A drug eluting poly(trimethylene carbonate)/poly(lactic acid)-reinforced nanocomposite for the functional delivery of osteogenic molecules
Source: Int J Nanomedicine. 2018 Sep 24;13:5701–18. doi: 10.2147/IJN.S163219 (PMC6161751; doi:10.2147/IJN.S163219)

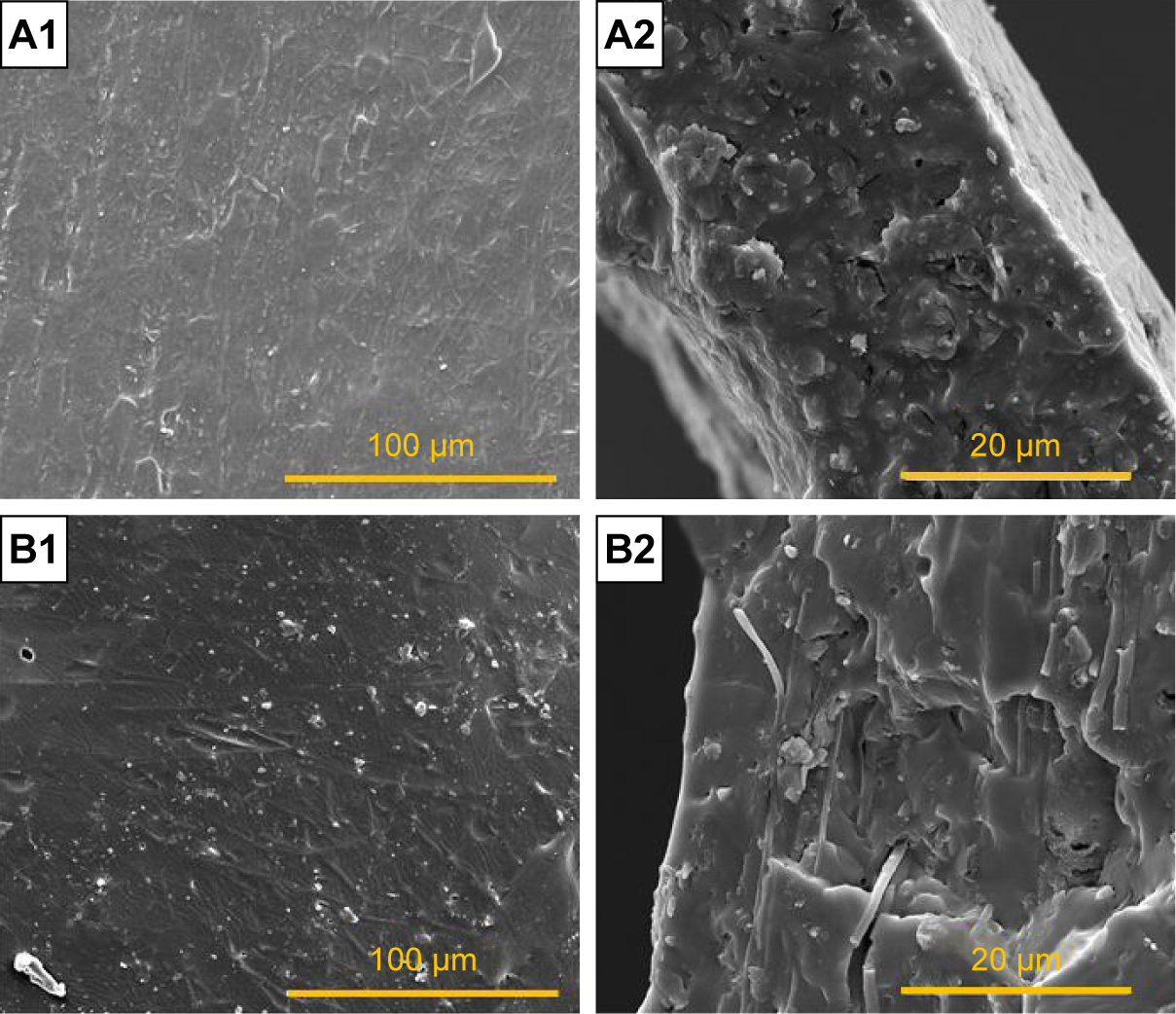

Supplement: Figure S1 — SEM images of PTMC/PLA fiber composites after 35 days in vitro release tests: PTMC/PLA 1 (A1) surface, (A2) cross-section and PTMC/PLA 2 (B1) surface, (B2) cross-section (scale bar 20 µm). Abbreviations: PLA, poly(lactic acid); PTMC, poly(trimethylene carbonate); SEM, scanning electron microscopy. [file ijn-13-5701s1.tif]

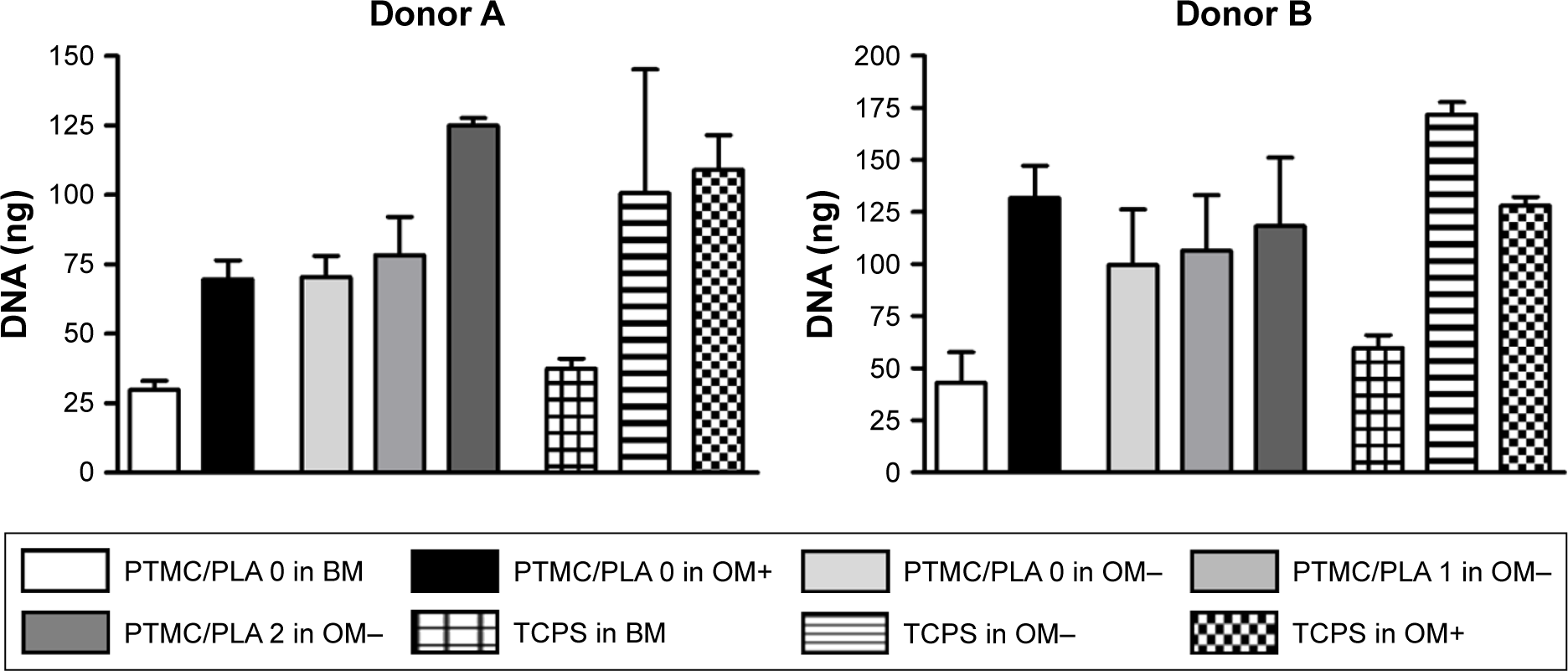

Supplement: Figure S2 — Cell quantification determined by DNA measurement of hBMSCs present on the different substrates in various media (BM, OM− and OM+) at Day 21, for two donors presented separately (in ng/film). Abbreviations: BM, basal medium; hBMSCs, human bone marrow mesenchymal stem cells; OM, osteogenic media; PLA, poly(lactic acid); PTMC, poly(trimethylene carbonate); TCPS, tissue culture polystyrene. [file ijn-13-5701s2.tif]

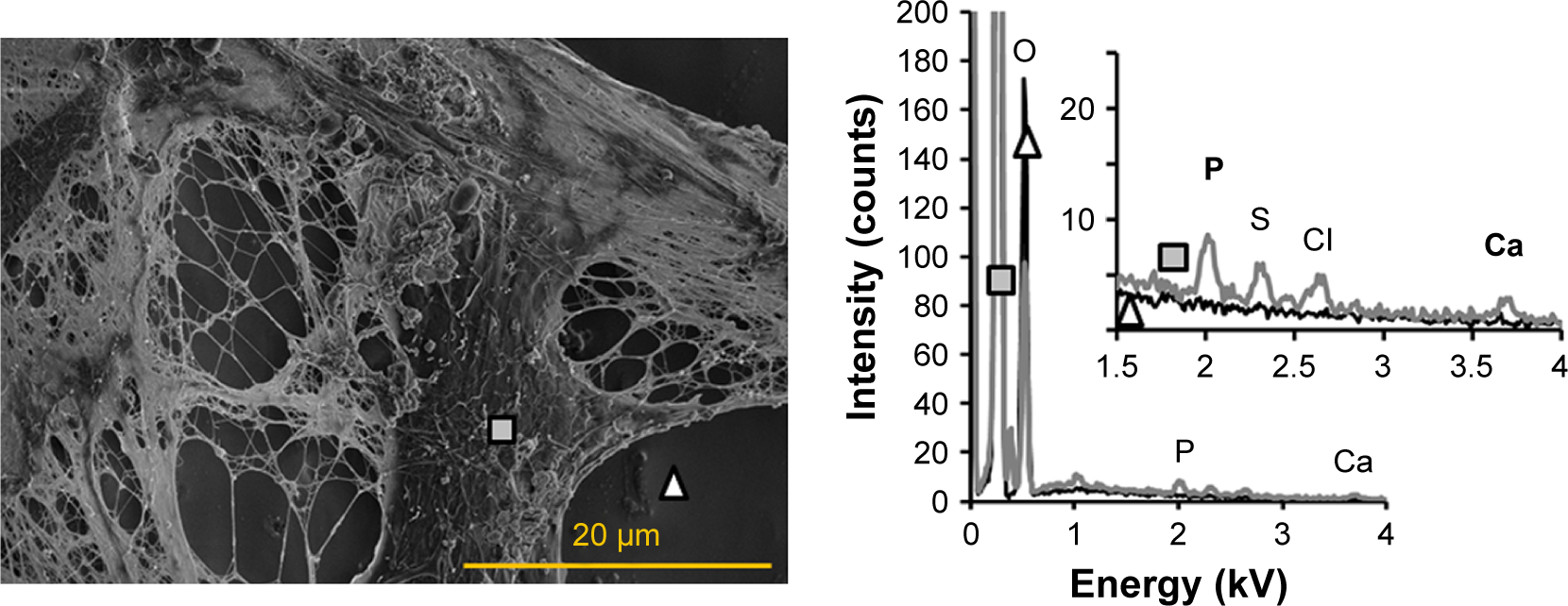

Supplement: Figure S3 — EDX analysis of biomineralization illustrated on sample PTMC/PLA 2 in OM− revealing the presence of Ca and P elements deposited in the pericellular environment (square) and its absence on cell-free area (triangle). This analysis was determined by energy-dispersive X-ray (EDX, Oxford Instruments, Abingdon, UK), following C coating. Abbreviations: EDX, energy dispersive X-ray; OM, osteogenic media; PLA, poly(lactic acid); PTMC, poly(trimethylene carbonate). [file ijn-13-5701s3.tif]
